# AutoBar: Automatic Barrier Coverage Formation for Danger Keep Out Applications in Smart City

**DOI:** 10.3390/s23187787

**Published:** 2023-09-10

**Authors:** Ying Shao, Qiwen Wang, Xingjian Lu, Zhanquan Wang, E Zhao, Shuang Fang, Jianxiong Chen, Linghe Kong, Kayhan Zrar Ghafoor

**Affiliations:** 1 Shanghai Technical Institute of Electronics and Information, Shanghai 201411, China; dzxshy@163.com (Y.S.); fangafs@163.com (S.F.); 20220088@stiei.edu.cn (J.C.); 20225018@stiei.edu.cn (L.K.); 2 Department of Computer Science and Engineering, Shanghai Jiao Tong University, Shanghai 200240, China; wqw1026@sjtu.edu.cn; 3 School of Computer Sciences and Technology, East China Normal University, Shanghai 200050, China; 4 School of Information Science & Engineering, East China University of Science and Technology, Shanghai 200231, China; zhqwang@ecust.edu.cn; 5 Aerospace Technology Holding Group Co., Ltd., Beijing 100070, China; bupt_zhaoe@126.com; 6 Department of Computer Science, Knowledge University, Erbil 44001, Iraq; mrkayhanz@gmail.com

**Keywords:** barrier coverage, mobile sensor networks, boundary detection, convex analysis, virtual force

## Abstract

Barrier coverage is a fundamental application in wireless sensor networks, which are widely used for smart cities. In applications, the sensors form a barrier for the intruders and protect an area through intrusion detection. In this paper, we study a new branch of barrier coverage, namely *warning barrier coverage* (WBC). Different from the classic barrier coverage, WBC has the inverse protect direction, which moves the sensors surrounding a dangerous region and protects any unexpected visitors by warning them away from the dangers. WBC holds a promising prospect in many danger keep out applications for smart cities. For example, a WBC can enclose the debris area in the sea and alarm any approaching ships in order to avoid their damaging propellers. One special feature of WBC is that the target region is usually dangerous and its boundary is previously unknown. Hence, the scattered mobile nodes need to detect the boundary and form the barrier coverage themselves. It is challenging to form these distributed sensor nodes into a barrier because a node can sense only the local information and there is no global information of the unknown region or other nodes. To this end, in response to the newly proposed issue of the formation of barrier cover, we propose a novel solution *AutoBar* for mobile sensor nodes to automatically form a WBC for smart cities. Notably, this is the first work to trigger the coverage problem of the alarm barrier, wherein the regional information is not pre-known. To pursue the high coverage quality, we theoretically derive the optimal distribution pattern of sensor nodes using convex theory. Based on the analysis, we design a fully distributed algorithm that enables nodes to collaboratively move toward the optimal distribution pattern. In addition, AutoBar is able to reorganize the barrier even if any node is broken. To validate the feasibility of AutoBar, we develop the prototype of the specialized mobile node, which consists of two kinds of sensors: one for boundary detection and another for visitor detection. Based on the prototype, we conduct extensive real trace-driven simulations in various smart city scenarios. Performance results demonstrate that AutoBar outperforms the existing barrier coverage strategies in terms of coverage quality, formation duration, and communication overhead.

## 1. Introduction

Barrier coverage [1] is one vital application in wireless sensor networks (WSNs) for smart cities [2,3], which forms sensor nodes surrounding barriers to protect a region by detecting all intruders. A wide range of safety scenarios of smart city demand barrier coverage exist, for example, the country border surveillance for stowaway detection. In this paper, we extend the classic concept of barrier coverage to a new branch, which moves the sensor nodes surrounding a dangerous region and protects any unexpected visitors by warning them away from the dangers, so-called *warning barrier coverage* (WBC). The WBC is promising in many danger keep out application for smart cities. For example, a WBC can surround debris areas in floods and alarm rescue workers to avoid unnecessary harms. Moreover, WBC can warn people to avoid entering dangerous areas such as hazardous gas leaks and even nuclear radiations in cities.

We compare the classic barrier coverage and WBC in Table 1. Different from the classic barrier coverage, WBC focuses on the danger keep out applications whose boundary of target region is previously unknown. To avoid danger, people cannot become close to the region or deploy sensor nodes manually. Hence, besides the visitor detection, sensor nodes in WBC should have the capability to detect the boundary as well. Based on the sensing results, mobile sensor nodes form the barrier collaboratively.

Regardless of the classic barrier coverage used in WBC, the formation process [4] is crucial because newly deployed sensor nodes lack a dependable infrastructure for communication and detection. The formation process in WBC is defined to form sensor nodes into *k* barriers enclosing the region, thus detecting or warning unexpected visitors.

Researchers have put forward various solutions in the literature for classic barrier coverage formation for smart cities. For instance, Kumar [1] proposed a centralized algorithm to determine weak *k*-barrier coverage in a region using randomly deployed sensor networks. Later, ref. [5] devised efficient algorithms to construct strong sensor barriers. And ref. [6] studied the barrier coverage of line-based deployment. In addition, ref. [7] funded a cluster-based barrier construction algorithm in mobile wireless sensor networks.

Different from the known region in classic barrier coverage, the boundary of a dangerous region in WBC is usually unknown. Hence, the classic formation approaches are not appropriate for WBC in smart cities. In addition, most existing works [1,6] fall into the category that forms the barrier coverage by stationary sensor nodes. Nevertheless, if the stationary sensor nodes are stochastically deployed, many redundant nodes will be needed to ensure a strong barrier [5]. On the other hand, if the stationary nodes are manually deployed, a significant amount of manpower and time are consumed. Especially in some cases for smart cities, the region is rather large in scale or hides dangers. Therefore, using stationary nodes is more of a hindrance than a help in WBC. A few state-of-the-art works also considered using mobile sensor nodes [8,9,10] to facilitate the barrier coverage. However, these works assumed that the region boundary is pre-known [11], which is not practical in WBC.

### 1.1. Challenge and Countermeasure

- To tackle the formation problem in WBC, in this paper, we propose a distributed movement strategy for mobile nodes to form the barrier coverage automatically for smart cities. The basic objective of the proposed strategy is to leverage the given number of mobile nodes to form a barrier coverage with the highest coverage quality, i.e., the maximal *k*-barrier coverage [1]. However, in scenarios wherein sensor nodes lack prior knowledge of their initial positions or the boundaries of the region, their movement can only be determined based on the local information they sense and communicate. This practical challenge presents a significant design obstacle.
- In order to address the challenge, we study the barrier coverage formation problem as follows: First, based on the convex analysis [12], we derive the following: *the optimal distribution pattern for k-barrier coverage is that all sensor nodes are evenly distributed on the convex hull of the region*. Second, we devise an algorithm *AutoBar*, which navigates the sensor nodes to detect the region boundary by themselves and then gradually move, approaching the optimal distribution pattern based on their local information. Third, extensive simulations verify the validity of AutoBar and evaluate its characteristics such as formation duration, communication overhead, and moving distance.

### 1.2. Major Contribution

- As far as we know, this is the first work to cover the coverage problem of the alarm barrier for smart cities, wherein the regional information is not pre-known.
- The optimal distribution of sensor nodes with maximum *k*-barrier coverage is derived to guide design.
- A fully distributed algorithm is developed to automatically form barrier coverage for sensor nodes.

The rest of this paper is organized as follows: Section 2 presents the related work. Section 3 formulates the barrier coverage formation problem. Section 4 investigates the optimal distribution pattern. The proposed algorithm is designed in Section 5 and the practical issues are discussed in Section 7. Section 6 depicts the results of simulation. In Section 8, we present conclusion and future work.

## 2. Related Work

In the construction of smart cities, information and communication technologies are used to improve the living standards and management of citizens and governments [13,14]. The Internet of Things (IoT) using sensors is widely used in smart cities [15]. In particular, the coverage-related problem [16,17] is a fundamental topic in WSNs to measure the monitoring quality of a sensor network deployed in a given region. Barrier coverage guarantees the detection of any intruder attempting to cross the barrier of sensor networks or penetrating the protected region. There are numerous studies on classical intrusion detection and avoidance [18,19,20]. Ref. [21] presents methods for intrusion detection and tracking with pan–tilt cameras. And ref. [22] proposes a probabilistic sensor tasking algorithm in which cameras sense the environment independently of one another, thus reducing the communication overhead. In addition, diverse directions are excellently studied for coverage problems, such as barrier coverage [1], sweep coverage [23], surface coverage [24], and trap coverage [25].

In these directions, barrier coverage is one valuable and practical application for smart cities, which is advocated in [1] for the purpose of intrusion detection in country borders, critical infrastructure protection, and battlefield perimeter surveillance. The barrier coverage formed by stationary nodes has been widely studied. For instance, the minimum cost for achieving *k*-barrier coverage is calculated in [26]. In [5], strong sensor barriers were devised. Line-based and curve-based barrier coverage were studied by [6,27], respectively. Multi-round sensor deployment for guaranteed barrier coverage is proposed in [28]. Nevertheless, a significant amount of resources such as redundant nodes in stochastic deployment and manpower cost in manual deployment will be needed due to the reliance on stationary nodes only.

Mobile nodes for barrier coverage was firstly introduced in [4], in which the nodes with limited mobility (e.g., one-step move with one chance) are utilized to improve the quality of barrier coverage. With the rapid development of autonomous robot technology, sensor nodes with strong mobility [29] become practical. In addition, a movement barrier formation algorithm MobiBar designed in [11] presented distributed algorithms for barrier coverage using sensor relocation. Ref. [30] proposed a heuristic target-barrier construction algorithm to solve the target-barrier coverage problem while satisfying the boundary constraint conditions. These works mainly focus on centralized analysis, which is not suitable for large-scale barrier coverage for smart cities.

Distributed algorithms for mobile barrier coverage were also investigated in the literature. The chain reaction algorithm [31] was firstly developed for mobile barrier formation. But it totally ignores the situation of node failure, which may lead to certain loopholes in the barrier. Based on mobility and intruder prior information, PMS [32] is able to improve the quality of barrier coverage. However, PMS assumes that the region knowledge is pre-known, which is not practical in most real WBC applications for smart cities. Moreover, ref. [33] presented a distributed cellular automaton based algorithm for the autonomous deployment of mobile sensors. The limitation is that the number of sensors needs to be deployed in a fixed manner. Our method focuses on this practical problem and is able to complete the barrier coverage without prior knowledge of regional information. In addition, we have listed references using Stationary nodes and Mobile nodes in Table 2.

Thus, it is essential to design a distributed, fault-tolerant, and automatic barrier coverage formation method using mobile nodes for WBC with the unknown regions for smart cities. The Autobar we proposed can be competent for this task.

## 3. Problem Formulation

In this section, we model the objects and then formulate the automatic barrier coverage formation problem.

### 3.1. System Model

*Region:* The region of interest *A* is the area needing to be surrounded by the barrier coverage. Assume that the region *A* is an enclosed area on a 2D plane, which has a continuous boundary in our model. We also assume that the detailed boundary information of the region *A* is not pre-known. The only known information is the general location of *A*. This is a practice-motivated assumption. For example, in a hazardous gas leakage incident of city sewage pipelines, the barrier coverage formation is strongly desired to surround the diffuse region rapidly and automatically, so that any unexpected visitor can be alarmed when she enters the dangerous region. In this case, the boundary of the diffuse region is not pre-known. And we can only obtain the general location where the hazardous gas leak happens.

**Figure 1:**
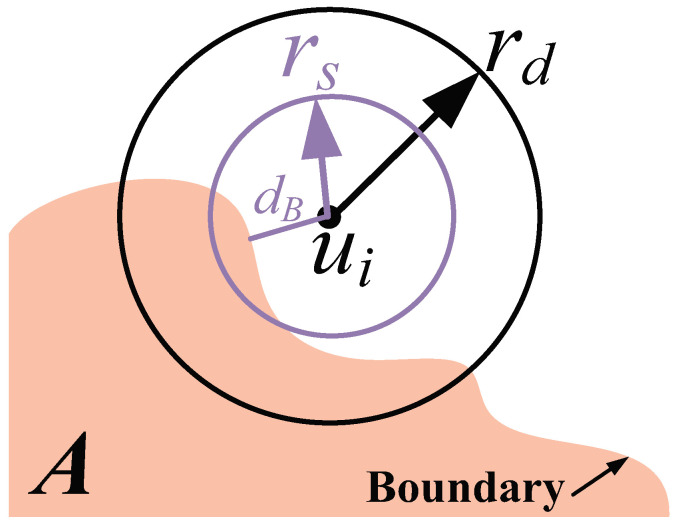
A node $u_{i}$ has two sensors to sense the region and detect unexpected visitors, respectively.

*Node:* In our model, a sensor node is denoted by $u_{i}$, where 1≤i≤n and *n* is a given number. Any node can move on the plane within the maximal velocity *v*. A node has at least two sensors to sense the region (e.g., the debris area, the nuclear area) and the unexpected visitors (e.g., the ships, the people), respectively. The sensing area of each sensor is assumed to be the widely adopted disk model [1]. The disk radius of the sensor for region sensing is denoted by $r_{s}$. Within $r_{s}$, we assume that the distance from the node to the region boundary $d_{B}$ can be obtained by this sensor (e.g., camera sensor) as shown in Figure 1. In addition, the disk radius of the other sensor for visitors detection is denoted by $r_{d}$. The communication range between nodes is $r_{c}$. Without loss of generality, we set $r_{c}>2r_{d}$ as the setting in [1]. Every node $u_{i}$ knows its location information by equipping it with auxiliary devices such as GPS. The autonomous robotic fish for debris monitoring [34,35] is an example of such sensor node, which can move in floods, has a camera to sense the debris, has an acoustic sensor to detect any approaching rescue workers, is equipped with GPS to know its location, and has a Zigbee module to communicate with other robotic fishes and to warn the approaching rescue workers.

**Figure 2:**
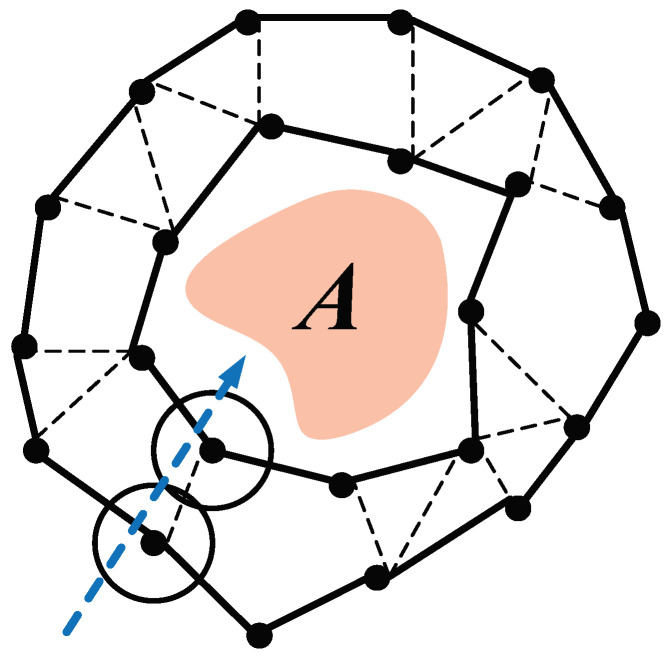
Coverage map of strong *k*-barrier coverage around area *A*, where *k* = 2.

*Warning Barrier coverage:* We use a graph G(n)=(V,E) to describe the warning barrier coverage as in [1]. The set *V* consists of the vertexes corresponding to the nodes. If the distance between any two vertexes is less than $2r_{d}$, then there is an edge between them. Strong barrier coverage [5] is described as a closed chain consisting of edges surrounding the region. Due to the mobility of nodes, this study only considers the formation of strong barrier coverage. Strong *k*-barrier coverage is expressed as *k* vertex-disjointed chains in G(n). In other words, any unexpected visitor that crosses the barriers would be detected by at least *k* nodes. We adopt *k* to measure the quality of barrier coverage in this paper. Figure 2 shows an example of a k=2 barrier coverage. Any path (e.g., the dash arrow) crossing the barrier coverage is covered by two nodes. Since the region *A* is not pre-known and the number of nodes *n* should be given before deployment, we practically set that *n* is a large enough number for barrier coverage formation to the region.

### 3.2. Fundamental Problem

The automatic barrier coverage formation (ABCF) problem for WBC is defined as *the automatic movement of all mobile nodes to form the maximum k barrier coverage around the region of interest*.

The goal of solving the ABCF problem is to maximize *k* under the constraints of given *n* nodes. This problem is non-trivial due to the following challenges: First, sensor nodes just have local information through communication and sensing. Without global information about boundary, it is difficult for nodes to know their destinations and paths. Second, it is common that some sensor nodes fail during the movement due to mechanical breakdown or depleted battery. Thus, the movement strategy should take the node failure into account. Third, centralized solutions consume heavy traffic on multi-hop transmissions, which are not appropriate for large-scale WBC for smart cities. Hence, a total distributed solution is required even if it is not easy.

## 4. Theoretical Analysis

In this section, when the region *A* and the number *n* are given, we export the maximum value *k*.

### 4.1. Different Types of *k*-Barrier Coverage

**Figure 3:**
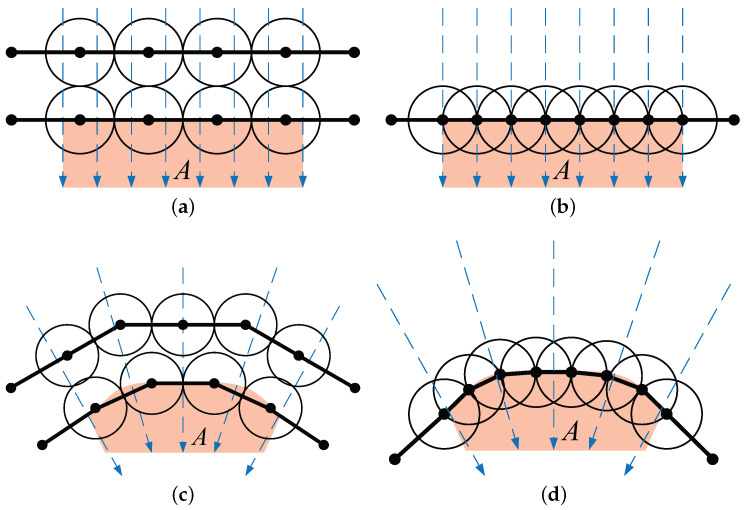
(**a**) Two-barrier coverage with multi-level type for a part of region with straight boundary. (**b**) Two-barrier coverage with line type for a part of region with straight boundary. (**c**) Two-barrier coverage with multi-level type for a part of region with arc boundary. (**d**) Two-barrier coverage with line type for a part of region with arc boundary.

In order to satisfy different coverage requirements for smart cities, the distribution of sensor nodes in *k*-barrier coverage has different types. Two typical types are as follows:

*Multi-level type:* All sensor nodes form several disjoint barriers [11]. Figure 3a shows that eight nodes form a two-barrier coverage with multi-level type for a part of the region with straight boundary.

*Line type:* All sensor nodes form several barriers on one line [6]. Figure 3b displays that eight nodes form a two-barrier coverage with line type for a part of the region with straight boundary.

In terms of the definition of *k*-barrier coverage [1], the value of *k* is only determined as the expected visitor being discovered by at least *k* nodes when it passes the barriers. In Figure 3a,b, no matter which crossing path (dash arrow) is selected, the path is always covered by two nodes. Hence, we observe that both types can provide k=2 barriers. The difference between two types is as follows: a visitor is detected by two nodes one after another in multi-level type. By contrast, it is detected by two nodes simultaneously in line type.

The advantage of multi-level type is to early detect the expected visitor benefiting from its “width”. Consequently, if the coverage requirement is to detect as early as possible, multi-level type is an excellent choice. On the contrary, the strength of line type is to form *k* barriers with the least number of nodes. Although two types achieve the same *k* in Figure 3a,b, if the straight boundary changes to be the arc boundary, in order to maintain k=2, multi-level type needs nine nodes, as seen in in Figure 3c, due to the outer ring always requiring more nodes, and line type only needs eight nodes, as seen in Figure 3d. In real scenarios for smart cities, an enclosed region with an arc boundary is a general case.

In this paper, the coverage requirement of the ABCF problem is to maximize *k* with the given *n* nodes. It is evident that the barrier of line type is a better candidate than the multi-level type.

### 4.2. Optimal Distribution Pattern

In this subsection, we derive the optimal destinations of *n* nodes in line type. Additionally, $f_{c}\left(A\right)$ denotes the length of the convex hull of region *A*. The method of obtaining $f_{c}\left(A\right)$ can be found in [12]. Therefore, we have

**Theorem** **1.** *To achieve the maximum value of k, it is sufficient for n nodes to be evenly distributed on the convex hull of region A. The maximal value of expectation k is*

(1) $$k=\frac{2nr_{d}}{f_{c}\left(A\right)}.$$

**Proof** **of** **Theorem** **1.** Convex analysis [12] establishes that the convex hull of a 2D region is the polygon with the smallest area and shortest perimeter that encompasses it. Thus, the shortest perimeter can be expressed as $f_{c}\left(A\right)$. This indicates that $f_{c}\left(A\right)$ represents the minimum length required for one barrier.

Due to an edge existing when $d\left(u_{i},u_{j}\right)\leq 2r_{d}$, where d() is the function to obtain the shortest distance between two objects, the maximum distance between any two connectable vertexes is $2r_{d}$. If we connect *n* vertexes in a series, the total length of all these vertexes is at most $2nr_{d}$.

Wrap the series of vertexes around the convex hull of region *A*, and we have the theoretical maximal value of $k=2nr_{d}/f_{c}\left(A\right)$.

Assuming a line-type distribution of sensor nodes along the shortest perimeter, each point on the perimeter is covered by multiple nodes. The minimum number of nodes covering a point is denoted as *k*, which corresponds to the definition of strong *k*-barrier coverage in [1].

When *n* vertexes are evenly distributed, we have the distance between any pair of the nearest neighbors

(2) $$\Delta =\frac{f_{c}\left(A\right)}{n}.$$

And any point on the perimeter is covered by at least

(3) $$k=\frac{2r_{d}}{\Delta }.$$

Combining Equations (2) and (3), we have

(4) $$k=\frac{2r_{d}}{\Delta }=\frac{2nr_{d}}{f_{c}\left(A\right)}.$$

The equality obtained in Equation (1) confirms the sufficiency of the condition, thereby proving Theorem 1. □

## 5. AutoBar Design

We have investigated that the destinations of *n* nodes are evenly distributed on the convex hull of the region in the last section. In this section, firstly, we present how the sensor nodes move to the destinations according to the proposed AutoBar algorithm. Secondly, we analyze the reasons behind the AutoBar design.

### 5.1. Design Overview

The source position of the sensor is assumed to follow random distribution in the basic problem, while the optimal distribution of the destination is derived. Then, in this section, we develop a cooperative algorithm for the sensors moving automatically from their source to the destination with only local information. To solve this problem, we exploit a distributed algorithm, called *AutoBar*, for the nodes with automatic movement from their initial positions to the destinations derived in Theorem 1 with only local information. The main procedure of AutoBar includes two steps: boundary seeking and barrier forming.

**Step 1: Boundary seeking.** The goal of this step is to design the moving path for a node to find the region boundary. Since the sensor nodes neither pre-know their initial positions (which depends on the deployment type) nor pre-know the location of the region *A* (no global information), the moving path cannot be pre-planed. To automatically form the barrier coverage, sensor nodes should find the region boundary in their first step no matter where their initial positions are. All initial positions can be classified into three cases: outside, on, and inside the region boundary.

**Figure 4:**
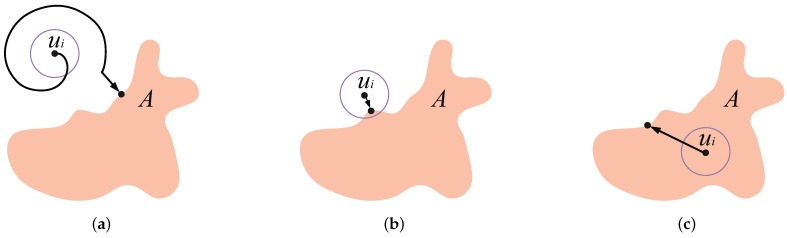
The paths for boundary seeking: (**a**) Nodes outside the region move along the boundary in a spiral pattern. (**b**) Nodes on the boundary remain on the boundary. (**c**) Nodes inside the region move directly toward the boundary.

*Case 1.1:* Outside the boundary. A node $u_{i}$ is considered outside the boundary if its distance from *A*, denoted as $d(u_{i},A)$, is greater than $r_{s}$. Node $u_{i}$ can self-determine whether it is outside the boundary through it cannot sense any part of region *A* within its $r_{s}$ disk, i.e., the sensing area of $u_{i}$ does not overlap with the region *A*. In this case, in order to find the boundary, this node is designed to randomly select an initial angle and then move along the Archimedean spiral, as shown in Figure 4a, until it meets the boundary of region *A*. The spiral path used for boundary detection in [36] offers several advantages. It evenly explores all directions, minimizing the risk of overlooking the region. Additional examination of the Archimedean spiral will be presented in the next subsection.

*Case 1.2:* On the boundary. When $d\left(u_{i},B\left(A\right)\right)\leq r_{s}$, the node $u_{i}$ is on the boundary, where B(A) is the function that obtains the boundary of region *A*. The node $u_{i}$ can be discretionary whether it is on the boundary by sensing the part of area of $u_{i}$ that overlaps with the region *A*. In this case, the current distance $d_{B}=d\left(u_{i},B\left(A\right)\right)$ can be sensed, and then the node moves along the shortest path to the boundary, as shown in Figure 4b.

*Case 1.3:* Inside the boundary. When $d\left(u_{i},B\left(A\right)\right)>r_{s}$ and $u_{i}\in A$, the node $u_{i}$ is inside the boundary. Node $u_{i}$ can be discretionary whether it is inside the boundary by sensing area of node $u_{i}$ completely overlaps with the region *A*, i.e., included by *A*. In this case, the node can move along a straight line after randomly selecting the direction to the boundary, as shown in Figure 4c.

**Step 2: Barrier forming.** After finding the boundary, the goal of the second step is for nodes to move together to form the maximum *k*-barrier coverage. Nodes have two directions along the boundary: clockwise (Right) and counterclockwise (Left). The nearest neighbor in any direction is called a direct neighbor. The barrier formation step is only based on the transmission of position information between close neighbors, which is easy to implement, energy-efficient, and little-delayed. Due to each node itself searching for boundaries, the situation of its neighbors is uncertain. There are three other cases of direct neighbors: 0, 1, or 2 direct neighbors.

**Figure 5:**
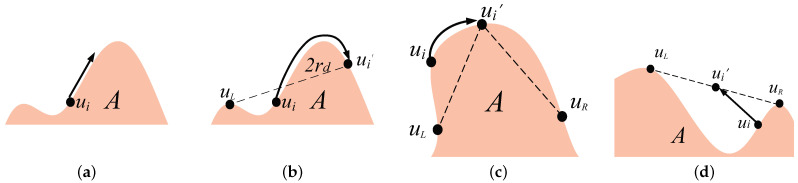
The movement strategy of the nodes for barrier forming: (**a**) If a node $u_{i}$ has no immediate neighbor, it selects clockwise direction and moves along the boundary until meeting the other node. (**b**) If a node $u_{i}$ has only one immediate neighbor, it moves away from that neighbor $u_{L}$ by a distance of $2r_{d}$ along the boundary. (**c**) If a node $u_{i}$ has two immediate neighbors and an internal angle ≤180°, it relocates on the boundary such that $d\left(u_{i}^{\prime },u_{L}\right)=d\left(u_{i}^{\prime },u_{R}\right)$. (**d**) When a node $u_{i}$ has two immediate neighbors and an internal angle >180°, it positions itself at the midpoint between $u_{L}$ and $u_{R}$.

*Case 2.1:* No neighbor. When there are no neighbors of the node $u_{i}$ in its sensing range $r_{d}$, it should self-move to find the other nodes to form the barrier together. We design that such node moves toward the clockwise direction, along the boundary of *A*, and with the velocity *v* until encountering the other node, as shown in Figure 5a.

*Case 2.2:* One direct neighbor. When a node $u_{i}$ has only one direct neighbor $u_{L}$ on one certain side, a virtual repulsive force [37] is generated between $u_{i}$ and $u_{L}$. This force leads to the node $u_{i}$ moving toward the opposite direction of $u_{L}$ and along the boundary of region *A* until the destination location that $d\left(u_{i},u_{L}\right)=2r_{d}$, as shown in Figure 5b.

*Case 2.3:* Two direct neighbors. When node $u_{i}$ on each side has two direct neighbors $u_{L}$ and $u_{R}$, these two neighbors will generate virtual forces. The amount of force depends on the distance $d(u_{i},u_{L})$ and $d(u_{i},u_{R})$. To balance these two forces, it demands that the destination location $u_{i}^{\prime }$ has the uniform distance to its direct neighbors $u_{L}$ and $u_{R}$, i.e., $d\left(u_{i}^{\prime },u_{L}\right)=d\left(u_{i}^{\prime },u_{R}\right)$. Moreover, a node with its two immediate neighbors can form an internal angle, which is facing the region. We have set a limit that the internal angle should not exceed $180^{o}$. In Figure 5c, the internal angle is α≤180°. Therefore, in order to balance the forces, the destination of $u_{i}$ is $u_{i}^{\prime }$ and the moving path is along the boundary. In Figure 5d, the inner angle is $\alpha >180^{o}$ because the part of the boundary is concave. In order to balance the forces and maintain the angle constraints, node $u_{i}$ moves directly to the bourn, which is the intermediate position between $u_{L}$ and $u_{R}$.

The pseudo-code of AutoBar is shown in Algorithm 1, where the lines 2–12 present the boundary-seeking step and the lines 13–28 present the barrier-forming step.
**Algorithm 1** AutoBar algorithm.*Executed on node $u_{i}$***Input:** the sensing range $r_{s}$,1:**while** $d\left(u_{i},u_{L}\right)\neq d\left(u_{i},u_{R}\right)$ **do**      // *step 1*2:     Sense within $r_{s}$ area and self-determine;3:     **switch** (Location)4:        **case** outside boundary: **do**5:           spiral_moving();6:           **break**;7:        **case** on boundary: **do**8:           direct_moving();9:           **break**;10:      **case** inside boundary: **do**11:         straight_moving();12:         **break**;    // *step 2*    //*$u_{i}^{\prime }$ is the next position to move, α is the inner angle*13:   Detect the direct neighbors $u_{R}$ and $u_{L}$;14:   Exchange position information with $u_{R}$ and $u_{L}$;15:   **switch** (Num of immediate neighbors)16:      ¡¡¡¡**case** 0: **do**17:         $u_{i}^{\prime }$←{clockwise direction with *v*};18:         ¡¡¡¡¡¡**break**;19:      ¡¡¡¡**case** 1: **do**20:         $u_{i}^{\prime }$←$\{d\left(u_{L},u_{i}^{\prime }\right)$=$2r_{d}$&$d(u_{i}^{\prime },B\left(A\right))$=0};21:         ¡¡¡¡¡¡**break**;22:      ¡¡¡¡**case** 2:23:         **if**(α>180) **do**24:            $u_{i}^{\prime }$← the middle position of $u_{R}$&$u_{L}$;25:         ¡¡¡¡¡¡**else do**26:            $u_{i}^{\prime }$←$\{d\left(u_{R},u_{i}^{\prime }\right)$=$d(u_{L},u_{i}^{\prime })$&$d(u_{i}^{\prime },B\left(A\right))$=0};27:         ¡¡¡¡¡¡**break**;28:   Move to $u_{i}^{\prime }$;29:**end while**

### 5.2. Design Analysis

We analyze our solution in depth in this section. Section 5.2.1 discusses the necessity of a distributed algorithm. This paragraph indicates the practice of a distributed algorithm in the barrier coverage formation problem. Section 5.2.2 studies the Archimedean spiral, which is adopted as the movement strategy in Case 1.1. We further derive the upper bound of time cost for the boundary-seeking step based on the theory of the Archimedean spiral. Section 5.2.3 explains the reason of the design strategies in Case 2.1 and 2.2, which involves the movement decision when a sensor has no or one immediate neighbor. Section 5.2.4 gives the design reason of the limitation of the internal angle. This design is the foundation to distributively realize the convex hull. Section 5.2.5 provides the reason behind the local balance design. Combined with the local balance design and the internal angle design, we prove that the maximal k-barrier coverage formation can be achieved. And Section 5.2.6 describes the chain reaction procedure of AutoBar. According to our design, AutoBar cannot move sensors to their final destination directly, but gradually approaches the optimal distribution.

#### 5.2.1. Necessity of a Distributed Algorithm

It is necessary that the proposed design can determine the movement strategy distributively due to the following reasons: First, since the communication range $r_{c}$ and the sensing range $r_{s}$, $r_{d}$ are not infinite of smart cities, any node cannot directly know the global information such as the real-time positions of all the other nodes and the boundary of region *A*. Second, if the nodes share their real-time positions by multi-hop transmission, the communication overhead is too high, especially when *n* is large. Thus, we design AutoBar, in which every node determines its movement only based on its local information.

#### 5.2.2. Archimedean Spiral in Case 1.1

In order to realize the automatic seeking, every node determines its movement according to Step 1. The movement strategies in Case 1.2 and 1.3 (shown in Figure 4b,c) are intuitive. Then, we discuss the moving path of the Archimedean spiral in Case 1.1.

**Figure 6:**
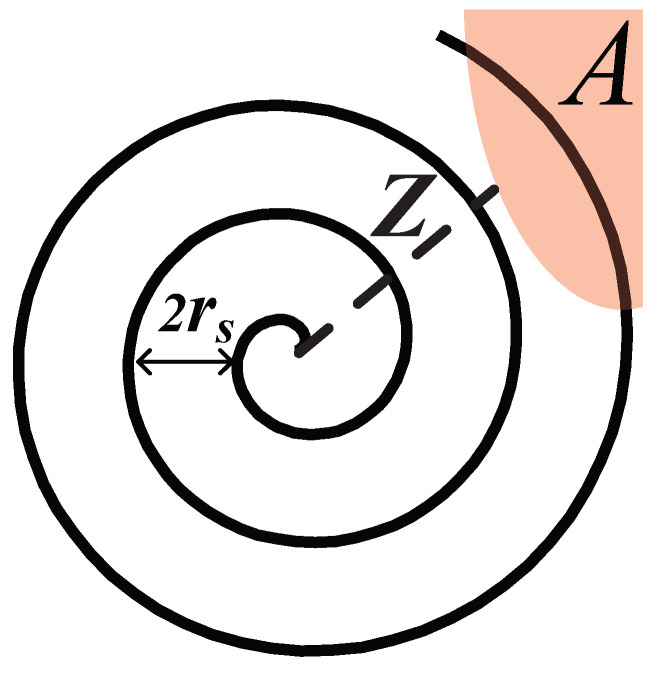
The Archimedean spiral with pitch $D=2r_{s}$.

The Archimedean spiral [38] is defined as the locus that rotates with constant angular velocity. One special property of such spiral is that its distance between successive turnings *D* (also known as pitch) is a constant. Hence, a node in Case 1.1 is set to move along the Archimedean spiral with setting $D=2r_{s}$, as shown in Figure 6. In polar coordinates (r,θ), this path can be described by the following equation:

(5) $$r\left(\theta \right)=\frac{D}{2\pi }\theta =\frac{r_{s}}{\pi }\theta .$$

Since any node deployed outside the boundary does not know the location of region *A*, the node moving along this spiral path can gradually expand the seeking scope. On one hand, due to its constant angular velocity definition, the node searches for boundaries with equal probability in all directions. On the other hand, the node will not miss any area even in a very small region *A* due to the constant pitch property $D=2r_{s}$. Thus, the successful seeking for region *A* is guaranteed.

**Theorem** **2.** *Given that Z is the shortest distance between the initial position of a node $u_{i}$ and the region A, the time cost $T_{s}$ for this node to seek the boundary of A is*

(6) $$T_{s}\leq \frac{2r_{s}\pi }{v}{\left\lceil \frac{Z}{2r_{s}}\right\rceil }^{2}+\frac{r_{s}}{2v\pi }\ln\left(4\pi \left\lceil \frac{Z}{2r_{s}}\right\rceil \right),$$

*if this node moves along the Archimedean spiral with pitch $2r_{s}$ using the velocity v.*

**Proof** **of** **Theorem** **2.** Since $d(u_{i},A)=Z$ and the pitch of the spiral is $2r_{s}$, we can obtain that the number of turnings, for which the node needs to move to find the region *A*, is at most $\left\lceil \frac{Z}{2r_{s}}\right\rceil$, as shown in Figure 6.

Hence, the moving distance *L* of this node is no more than the length of these $\left\lceil \frac{Z}{2r_{s}}\right\rceil$-turning spiral. Using the theory of Archimedean spiral [38], we obtain

(7) $$L\leq \int_{0}^{2\pi \lceil \frac{Z}{2r_{s}}\rceil }\sqrt{r{\left(\theta \right)}^{2}+r^{\prime }{\left(\theta \right)}^{2}}d\theta .$$

Substitute Equation (5) into Equation (7), we obtain

(8) $$L\leq \frac{r_{s}}{\pi }\int_{0}^{2\pi \lceil \frac{Z}{2r_{s}}\rceil }\sqrt{\theta^{2}+1}d\theta .$$

In terms of the integral formula in [39], we further obtain

(9) $$L\leq \frac{r_{s}}{\pi }\left(\frac{1}{2}\theta \sqrt{\theta^{2}+1}+\frac{1}{2}\ln\left|\theta +\sqrt{\theta^{2}+1}\right|\right){|}_{0}^{2\pi \lceil \frac{Z}{2r_{s}}\rceil }.$$

Approximate $\sqrt{\theta^{2}+1}$ by θ, we simplify Equation (9) to

(10) $$L\leq 2r_{s}\pi {\left\lceil \frac{Z}{2r_{s}}\right\rceil }^{2}+\frac{r_{s}}{2\pi }\ln\left(4\pi \left\lceil \frac{Z}{2r_{s}}\right\rceil \right).$$

Substitute Equation (10) into $T_{s}=L/v$, the result of Theorem 2 is obtained. □

#### 5.2.3. Movement Strategy of Case 2.1 and 2.2

After the boundary seeking, a node needs to move for barrier-forming based on the number of immediate neighbors.

When a node has no immediate neighbor, it implies that this is an isolated node. In order to form a barrier coverage, this node has to find the other nodes. Thus, Case 2.1 proposes that the isolated node selects the clockwise direction and moves with its maximal velocity *v* to encounter one node as soon as possible. Moreover, in an extreme scenario, if only one node is utilized, this node will keep moving along the boundary of *A* according to Case 2.1, which is equivalent to providing a sweep barrier coverage for *A*.

When a node has only one immediate neighbor, it indicates that this node is at the side of a segmental barrier, which certainly covers a partial boundary of the region. In order to form a complete barrier, the segmental barrier is desired to cover the boundary as much as possible. Hence, Case 2.2 proposes that the virtual repulsive force pushes this node running away from neighbors and maintaining the distance at less than $2r_{d}$. This strategy is able to enlarge the length of this segmental barrier, cover more vacant boundaries, and maintain the connection of the segmental barrier. Such node will keep moving until it encounters the other node as its second immediate neighbor; then, they can move according to Case 2.3 and form the *k*-barrier coverage gradually.

#### 5.2.4. Limitation of Internal Angle in Case 2.3

The movement strategy in Case 2.3 is actually the main force to shape the *k*-barrier coverage. Recall that the maximal *k*-barrier coverage is achieved when *n* nodes are *evenly* distributed on the *convex hull* of *A* with respect to Theorem 1. In Case 2.3, we propose the limitation of internal angle to ensure the convex hull and design the local balance to realize the even distribution of sensor nodes.

A formed barrier is the convex hull of the region *A* which can be determined by (i) *a convex shape* with (ii) *the shortest perimeter* to cover the region. Hence, we set the limitation of internal angle α≤180° in Case 2.3 to satisfy these two conditions.

For condition (i), since every internal angle is limited by α≤180°, the formed barrier is an obvious convex shape.

For condition (ii), when the partial segment of the boundary is convex, the node forms the barrier along the boundary, as shown in Figure 5c. In the case of convex segment, the boundary itself is the shortest radian [12]. On the other hand, when the partial segment of the boundary is concave, the nodes form a straight barrier for ensuring the limitation of internal angle, as shown in Figure 5d. In the concave case, the straight line definitely presents the shortest distance. Consider all convex/concave segments of the boundary together, and the entire formed barrier still has the shortest perimeter.

Hence, the limitation of internal angle can ensure that the nodes form the convex hull.

#### 5.2.5. Local Balance in Case 2.3

Only forming the convex hull is not adequate to tackle the maximal *k*-barrier forming; therefore, another key question is: how does a node locally determine whether all nodes are *evenly* distributed on the convex hull?

In [1], Kumar et al. confirmed that no distributed algorithm can payoff whether the barrier coverage is formed in a stationary scenario. However, in our mobile node scenario, we propose the local balance criterion to determine the achievement of *k*-barrier coverage.

**Definition** **1.** *Local balance: The distance between a sensor node and its two immediate neighbors is equal.*

**Theorem** **3.** *The maximal k-barrier coverage is achieved when each sensor node satisfies the local equilibrium.*

**Proof** **of** **Theorem** **3.** Due to the limitation of internal angle, all nodes are on the convex hull, which is a closed chain. Thus, when every node satisfies the local balance, the distance between any pair of immediate neighbors is consequentially isometric, which meets the optimal distribution pattern in Theorem 1. Therefore, the maximal *k*-barrier coverage is achieved. □

Therefore, the local balance criterion can ensure that the nodes *evenly* distribute on the convex hull in regard to Theorem 3.

This theorem also demonstrates that a distributed algorithm to *k*-barrier coverage determination can be feasible in a mobile scenario, because every node can locally determine it by the local balance criterion and obtain the value of *k* by the distance Δ according to Equation (3).

Furthermore, the local balance is also set to be the terminating condition, as shown in the line 1 of the AutoBar algorithm in Algorithm 1. This implies that a node will stop moving only if its local balance is achieved.

#### 5.2.6. Chain Reaction Procedure of AutoBar

Based on the above design analysis, it is obvious that AutoBar is a chain reaction procedure, where the nodes do not reach the final destinations directly, but gradually approach the optimal distribution pattern.

In Step 1, since the initial positions of the nodes are unknown, these nodes consume a different duration on boundary seeking. We adopt a first arrive first work (FAFW) mechanism, i.e., once a node finds the boundary, it starts Step 2 immediately without waiting for other nodes. This mechanism has two advantages: First, a node does not need to know the information from all the others, which meets the requirement of a distributed algorithm. Second, this mechanism assists to form at least a one-barrier coverage as fast as possible, which is valuable in certain time-critical applications.

In Step 2, a partial number of nodes may have built a barrier coverage firstly due to the FAFW mechanism. Then, any new arrival node will change the relationship of immediate neighbors and break the current local balance. Consequently, all the other nodes on the barrier should restart to adjust their positions little by little until the re-achievement of their (including the new arrival node together) local balance. This chain reaction repeats until all *n* nodes join into the barrier, and then the automatic maximal *k*-barrier coverage formation is finally realized.

#### 5.2.7. Node Failure

AutoBar supports to re-form the barrier coverage when some nodes are failed. Assume a node is failed (damage or battery drained); the relationship of immediate neighbors will be changed and the local balance will be broken as well, which is similar to the new arrival node case. Then, the chain reaction will carry out to achieve the local balance of the rest nodes.

## 6. Performance Evaluation

In this section, we conduct extensive simulations in the scenario for smart cities to evaluate the proposed AutoBar algorithm.

### 6.1. Simulation Settings

**Figure 7:**
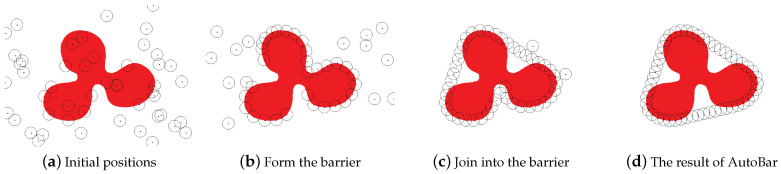
Four snapshots formed by AutoBar algorithm’s automatic barrier coverage when the initial positions of sensor nodes are randomly distributed in the region.

In our simulation, the region of interest *A* is the colored area in an 800 m × 600 m rectangle area, as shown in Figure 7a, and the other part is white. Assume the initial positions of all nodes should be in the rectangle area. Any sensor node is able to detect the boundary of the region (by distinguishing the color in our simulation) within its sensing range $r_{s}=25$ m. The range for visitor detection is also set to $r_{d}=25$ m. Thus, any node is depicted as a point with a radius of 25 m, as shown in Figure 7a. Nodes also can propagate information within their communication ranges $r_{c}=60$ m. In addition, the number of nodes is set as n=50 by default. Every node can move within the velocity of 10 m/s (even go outside the rectangle area). A node transmits its location information to its immediate neighbors once per second. The results described in this section are averaged over 100 simulation runs.

### 6.2. Performance Analysis

#### 6.2.1. Validation with Different Deployment Types

The purpose of the first simulation is to verify the universal validation of the proposed AutoBar algorithm on automatic barrier coverage formation (ABCF).

The initial positions of the nodes are not pre-known, which rely on the deployment types. In order to test the feasibility of AutoBar with diverse distributions of initial positions, we conduct this simulation based on two typical deployment types: (i) *Random* deployment, in which all nodes are uniformly scattered in the rectangle area; and (ii) *OnePosition* deployment, in which all nodes are deployed at one position.

Figure 7a–d are four snapshots using AutoBar in Random deployment type. Since the initial positions of all nodes are uniformly distributed, as shown in Figure 7a, the nodes need to seek the boundary of the given region according to Step 1 in AutoBar. Once some nodes (Case 1.2 and 1.3) detect the boundary, they start Step 2 to form the barrier directly, as depicted in Figure 7b. Figure 7c illustrates that some nodes join into the barrier after spiral moving (Case 1.1). And Figure 7d displays the result of AutoBar, which forms a strong k=2 barrier coverage.

**Figure 8:**
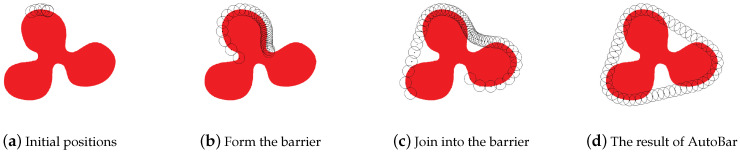
Four snapshots formed by AutoBar algorithm’s automatic barrier coverage when all sensor nodes are scattered at the same position at the beginning.

Correspondingly, Figure 8a–d are four snapshots using AutoBar in OnePosition deployment type, which is a simple and practical deployment type specialized for ABCF problem. We assume that all nodes are deployed at one position on the region boundary. Thus, the nodes can skip Step 1, and gradually enlarge the barrier according to Case 2.2. Figure 8d exhibits that AutoBar also achieves the same two-barrier coverage formation as it does in Random deployment.

Therefore, this simulation demonstrates that AutoBar is valid for ABCF problem though the initial positions of the nodes are different.

#### 6.2.2. Different Algorithms Comparison

This simulation evaluates the quality of the barrier coverage, i.e., the number of barriers *k* compared with the state-of-the-art algorithm MobiBar [11].

MobiBar is also a distributed algorithm to form the barrier coverage. One difference is that the goal of MobiBar is to form the *multi-level* barriers, as the example shown in Figure 3c. Another difference is that MobiBar works under an assumption of *pre-knowing* the region of interest. Under this assumption, the nodes do not need to seek the boundary and thus its application range is constrained. For fair comparison, we only compare AutoBar and MobiBar on the value of *k*, which just involves the final formed barriers. The comparison involving the boundary-seeking step such as formation duration, communication overhead, and moving distance are omitted.

**Figure 9:**
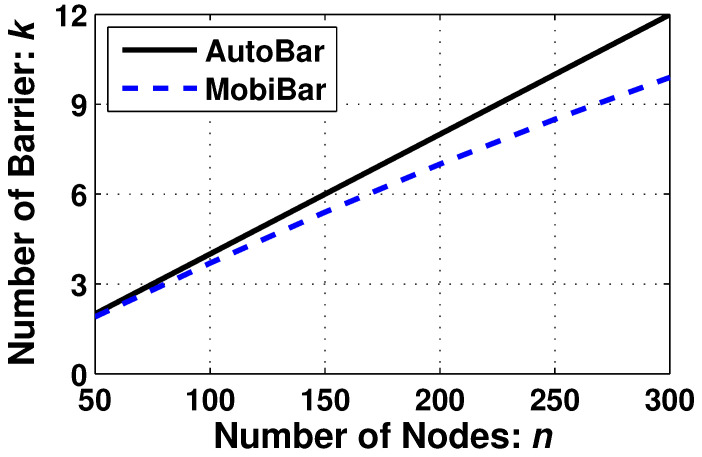
Comparison between AutoBar and MobiBar on the performance of *k* when varying *n*.

Figure 9 displays the performance of *k* while the number of nodes *n* vary from 50 to 300. Both algorithms show that *k* is proportional to *n*. However, our AutoBar achieves a little higher *k* than MobiBar. For example, when 300 nodes are utilized, AutoBar achieves 12-barrier coverage while MobiBar achieves 10-barrier coverage. This result proves that AutoBar fully utilizes the number of nodes to shape the maximal *k*-barrier coverage.

#### 6.2.3. Performance on Formation Duration

**Figure 10:**
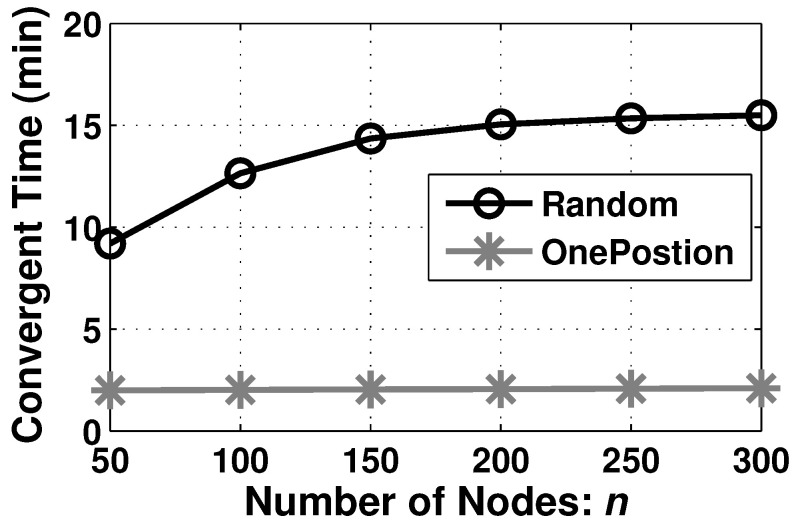
The formation duration of AutoBar in different deployment types when varying *n*.

Except for the quality of barrier coverage *k*, the formation duration is another significant metric to evaluate the proposed algorithm. A short duration is desired for ABCF. Hereby, we measure the formation duration of AutoBar in different deployment types in Figure 10.

In the OnePosition deployment type, all time is consumed by the Step 2 barrier forming, which does not need the Step 1 boundary seeking. Hence, the formation duration mainly depends on how long the nodes at two ends can encounter each other, as shown in Figure 8a–c. And thus, the shape of the region *A* and the velocity of nodes *v* determine the formation duration without the influence from the number of nodes *n*. As a result, the formation duration remains at 2 min when *n* varies from 50 to 300, as seen in Figure 10.

By contrast, in the Random deployment type, since several faraway nodes require much time to find the region boundary through the spiral path, the formation duration mostly relies on how long the last node can join into the barriers. When more nodes are utilized, it has a higher probability of a longer distance between the region and the initial position of the farthest node. Therefore, the formation duration of the Random deployment is increased from 9.5 to 15.5 min with the increase in *n*, as shown in Figure 10. In addition, the curve converges around 15.5 min because the longest distance between *A* and the farthest node (has to be deployed inside the rectangle area) is finite.

#### 6.2.4. Performance on Communication Overhead

**Figure 11:**
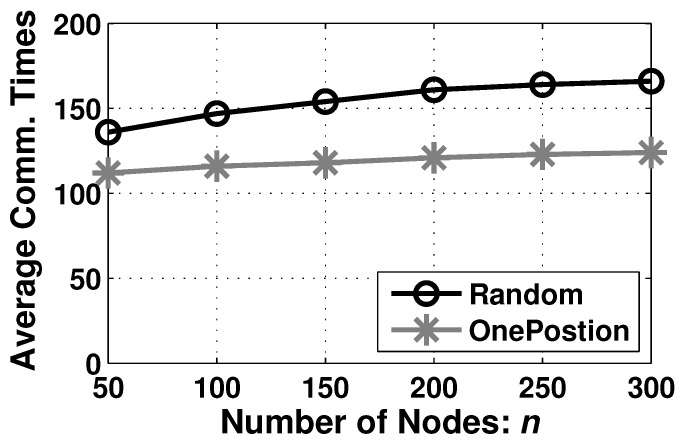
The communication overhead of AutoBar in different deployment types when varying *n*.

Moreover, we consider the average communication times of every node for ABCF in Figure 11. In AutoBar, the communication happens only in the barrier-forming step, which demands the location information from the immediate neighbors. As a result, in the OnePosition deployment type, the number of average communication times of every node is around 120. In addition and in the Random deployment type, the average communication times changes from 141 to 163 when *n* is increased from 50 to 300, whose tendency is similar to the formation duration curve in Figure 10. We find that the communication overhead is very low, so the current wireless devices are sufficient to transmit necessary information to perform AutoBar.

#### 6.2.5. Performance on Moving Distance

A mobile node is constrained by its equipped energy, which can support only the limited moving distance. The goal of this simulation is to measure the moving distance of every node using AutoBar in Random and OnePosition deployment types. The maximal, the average, and the minimal moving distances with different *n* are listed in Table 3. We find that the nodes do not move a long way. For example, even we randomly scatter 300 nodes, a node moves at most 9054 m and the average moving distance is 972 m. As another example, in the OnePosition deployment, no matter how many sensor nodes are utilized, the average moving distances remain around 350 m. These results further demonstrate the feasibility of AutoBar because current mobile nodes can satisfy the movement requirement.

Additionally, in terms of the above simulations, we strongly suggest to combine the OnePosition deployment type and AutoBar algorithm together to tackle the problem of automatic *k*-barrier coverage formation, whose advantages include simple deployment, high quality, quick formation, low overhead, and energy-saving.

## 7. Practical Issue

In this section, we discuss a few practical issues for smart cities when implementing AutoBar on mobile sensor nodes which include the mobility capability, energy consumption, computation and communication overhead, reliability and dependability, authentication and privacy, as well as hardware affordability and Scalability.

### 7.1. Mobility Capability

The proposed AutoBar demands that the nodes have the mobility capability. Plenty of mobile sensor nodes have been exploited in real applications for smart cities. For instance, in CarTel [29] project, vehicles equipped with sensors move along the roads. In addition, the SUMMIT [40] is a widely adopted multi-sensor robot, which can move on the diverse terrain. Furthermore, the Waalbot [41] robot is able to climb on the wall and ceiling. Hereby, using mobile nodes to form barrier coverage automatically has become practical recently for smart cities.

### 7.2. Energy Consumption

The energy consumption is another significant practical issue, which is considered by most WSN studies in smart cities. Different from the static WSNs, in the mobile scenario, movement costs much more energy than communication and computation [29]. The energy issue can be addressed in AutoBar due to the following reasons. On one hand, with the powerful battery, current autonomous robots such as the SUMMIT [40] can move 240 min with the velocity of 3 m/s, i.e., totaling 43.2 km. On the other hand, the sensor node using AutoBar is not required to move a long distance. For example, under our simulation setting in Section 6, the result shows that a node moves no more than 10 km.

### 7.3. Computation and Communication Overhead

The third issue needing to be considered is whether the computation and communication capability of sensor nodes are sufficient to perform AutoBar. From the algorithm in Algorithm 1, a node making one movement decision requires that the computational complexity is O(1) and the communication overhead is one message from two immediate neighbors, respectively. Current off-the-shelf mobile nodes such as the SUMMIT [40] (Embedded PC and WiFi) are more than adequate to apply our design.

### 7.4. Reliability and Dependability

Compared with the existing methods, AutoBar is totally distributed and is able to tolerate node failure. As mentioned in Section 5.2.7, Autobar has the ability to re-form the barrier coverage when some nodes fail. When a node fails, its neighbors can be regarded as “newly arrived nodes”, and a new local balance is reached through the chain reaction. This guarantees the reliability and dependability of AutoBar, which is fault-tolerant.

### 7.5. Authentication and Privacy

AutoBar can be widely used in smart cities, but it also brings problems worthy of attention like authenticity (people could be authenticated) and privacy (that have to be preserved). AutoBar is a distributed and automatic barrier coverage formation method using mobile nodes for WBC with the unknown regions. The main purpose of using AutoBar in smart cities is to keep humans out of dangerous areas such as nuclear leaks. An immediate warning can be issued for any unexpected visitors entering the covered region. It is possible to identify humans entering a particular area, and the technology itself can protect privacy [42,43].

### 7.6. Hardware Affordability

Deploying sensors without motion ability may cause a waste of resources. In addition, in a realistic case, the region of interest is usually large in scale and the task is dangerous and dull. It is impossible to deploy all sensors manually. Therefore, this paper introduces a solution that mobile sensors relocate their positions and self-organize the barrier coverage for the region. This design of AutoBar greatly reduces the cost of creating and deploying nodes.

### 7.7. Scalability

According to our design, AutoBar does not move sensors to their final destination directly, but gradually approaches the optimal distribution. In smart city applications, when the coverage region increases, we add new nodes. At this point, all the other nodes on the barrier should restart to adjust their positions until the re-achievement of their local balance. Ultimately, the automatic maximal *k*-barrier coverage formation can be achieved.

## 8. Conclusions

This article focuses on the application for smart cities of early warning barrier coverage and solves the problem of mobile nodes automatically forming barrier coverage. An AutoBar algorithm is proposed for nodes to automatically move nodes from their initial positions to form the maximum *k*-barrier coverage. The pragmatic algorithm can be widely used for different inception states, such as tatted or arbitrary deployment. AutoBar can warn people to avoid harmful gas leaks, nuclear contamination, and other dangerous areas in cities, reducing unnecessary injuries. To a certain extent, it also saves labor costs. It can effectively improve the safety and efficiency in smart cities, and has practical impact and extensive application value.

Due to the newly proposed issue of the formation of barrier cover, there are several promising research directions for the studies in the future. One of these directions is to extend AutoBar from an ideal 2D plane to a complex terrain situation for smart cities. For example, a surface with obstacles or holes. Second, optimizing the barrier coverage formation from other metrics such as the shortest formation time or the shortest moving distance is another are of research worth pursuing.

## Figures and Tables

**Table 1:** Comparison between classic barrier coverage and warning barrier coverage.

Barrier Coverage	Classic	Warning
Target Region	Known	Unknown boundary
Sensing Capability	Intruder detection	Visitor detection and boundary detection
Moving Capability	Mobile/static node	Mobile node
Typical Application	Border surveillance	Danger, keep out!

**Table 2:** Comparison table of some references.

Classification	Title	Publish Time
Stationary nodes	Constructing sensor barriers with minimum cost in wireless sensor networks [26].	2012
	Strong barrier coverage in directional sensor networks [5].	2012
	Barrier coverage with line-based deployed mobile sensors [6].	2013
	Curve-based deployment for barrier coverage in wireless sensor networks [27].	2014
	A multi-mode sensor management approach in the missions of target detecting and tracking [28].	2019
Mobile nodes	Barrier coverage with sensors of limited mobility [4].	2010
	Cartel: a distributed mobile sensor computing system [29].	2006
	Mobibar: Barrier coverage with mobile sensors [11].	2011
	Distributed algorithms for barrier coverage using relocatable sensors [30].	2013
	Automatic barrier coverage formation with mobile sensor networks [31].	2010
	Mobility and intruder prior information improving the barrier coverage of sparse sensor networks [32].	2013
	A distributed cellular automaton algorithm for barrier formation in mobile sensor networks [33].	2019

**Table 3:** Moving distance (meters) in different deployment types.

Num. of Nodes *n*		50	100	150	200	250	300
Random	Max	6057	7524	8463	8971	9009	9054
	Ave	774	851	910	946	963	972
	Min	17	13	10	8	6	6
OnePosition	Max	734	731	728	725	723	720
	Ave	351	349	348	346	345	344
	Min	0	0	0	0	0	0

## Data Availability

Data available upon request.
